# Infants’ and Toddlers’ Language, Math and Socio-Emotional Development: Evidence for Reciprocal Relations and Differential Gender and Age Effects

**DOI:** 10.3389/fpsyg.2020.580297

**Published:** 2020-11-23

**Authors:** Pauline L. Slot, Dorthe Bleses, Peter Jensen

**Affiliations:** ^1^Department of Clinical Child, Family and Education Studies, Utrecht University, Utrecht, Netherlands; ^2^School of Communication and Culture, Aarhus University, Aarhus, Denmark; ^3^TrygFonden’s Centre for Child Research, Aarhus University, Aarhus, Denmark; ^4^Department of Economics and Business Economics, Aarhus University, Aarhus, Denmark; ^5^Centre for Integrated Register-based Research, CIRRAU, Aarhus University, Aarhus, Denmark

**Keywords:** language, mathematics, socio-emotional, within and cross-domain development, toddler

## Abstract

Toddlerhood is characterized by rapid development in several domains, such as language, socio-emotional behavior and emerging math skills all of which are important precursors of school readiness. However, little is known about how these skills develop over time and how they may be interrelated. The current study investigates young children’s development at two time points, with about 7 months in between, assessing their language, socio-emotional and math language and numeracy skills with teacher ratings. The sample includes 577 children from 18 until 36 months of age of 86 childcare classrooms. The results of the autoregressive path analyses showed moderate to strong stability of language, socio-emotional and math language and numeracy skills, although the magnitude of associations was smaller for the latter. The cross-lagged path analyses highlighted the importance of language and socio-emotional skills for development in the other domains. Differential relations were found for the autoregressive and cross-lagged paths depending on gender and age. Language skills appeared a stronger predictor of boys’ socio-emotional and math language and numeracy skill development compared to girls. Girls’ socio-emotional skills predicted growth in math. For boys, socio-emotional and math language and numeracy skills appeared to be unrelated. Language skills showed stronger relations with the development of math language and numeracy skills for younger children as compared to older children. Also, for older children math language and numeracy skills negatively predicted growth in their socio-emotional skills. The findings provide more insights in how language, math language and numeracy skills and socio-emotional skills co-develop in the early years and as such have important implications for interventions aimed to support children’s development.

## Introduction

In order to succeed in school, children need a variety of skills, including pre-academic skills, such as basic language and math skills, socio-emotional and self-regulation skills (Rimm-Kaufman et al., 2009), often referred to as “school readiness” (e.g., Duncan et al., 2007; Justice et al., 2009). Early childhood is a period characterized by rapid growth of foundational cognitive, social and emotional skills (Shonkoff and Phillips, 2000). This has led to increasing attention and support for school readiness in preschool years (Duncan et al., 2007), mainly in view of early intervention to provide all children with the best start in school (e.g., Clements and Sarama, 2011; Melhuish, 2011; Magnuson and Duncan, 2016). Increasing evidence, however, suggests that disparities related to children’s socioeconomic status (SES), ethnicity or home language develop already at an earlier age, before preschool (for reviews see Denham et al., 2003, 2009; Dickinson et al., 2003; Clements and Sarama, 2011; Hoff, 2013; Purpura and Reid, 2016). In addition, increasing evidence has shown bidirectional relations between children’s pre-academic and socio-emotional skills, suggesting that less well developed socio-emotional skills impact children’s pre-academic development and vice versa, in preschool and beyond (Trzesniewski et al., 2006; Bornstein et al., 2013). Moreover, these skills are vital for children’s well-being in the here and now (Pollard and Lee, 2003; McAuley and Rose, 2014) by contributing to positive interactions with peers and (professional) caregivers while exploring their environment and during play (e.g., Denham and Brown, 2010; Clements and Sarama, 2014; Franzén, 2015). Therefore, more research is needed to better understand the development of these early skills, as important indicators of well-being in the here and now and as precursors of school readiness, and how these skills are interrelated in infant and toddler years.

### Early Pre-cursors of School Readiness

School readiness can be conceptualized along two important domains, including pre-academic skills, such as language and math, and socio-emotional skills, such as regulating and managing one’s emotions and engaging in social interactions (e.g., Denham, 2006; Duncan et al., 2007; Justice et al., 2009; Oades-Sese et al., 2011; Nix et al., 2013). Early language skills in particular, have shown to be of importance for the development of subsequent language skills (Whitehurst and Lonigan, 1998; Tomblin et al., 2000; Scarborough, 2001; Storch and Whitehurst, 2002; Morgan et al., 2015; Bleses et al., 2016). For example, Lee (2011) showed that the expressive vocabulary size at age 2 years predicted subsequent language achievement up to fifth grade. Another study showed predictions of infants’ vocabulary skills about 5 years later (Duff et al., 2015). Finally, a study by Bornstein et al. (2016) highlighted language stability from 15 months up to 11 years of age.

Also, the acquisition of mathematical competencies that are relevant for later math achievement takes place in the first years (e.g., Sarama et al., 2012; Clements and Sarama, 2014). Early math refers to a broad range of aspects, including counting, quantity, spatial relations, measurement and patterns (ibid, 2014). The learning trajectories introduced by Clements and Sarama (2014) illustrate both verbal (e.g., chanting numbers) and non-verbal skills (e.g., correspondence) in the development of math. Also Purpura and Reid (2016) highlighted the importance of “math talk” which includes words referring to quantities (e.g., “more,” “less”), spatial language (e.g., “above” or “beneath”) or names of shapes (e.g., “triangle” or “square”). Most research into math development included work on preschoolers or kindergartners. For instance, preschool children’s math abilities have shown to be predictive of fifth grade achievement (Nguyen et al., 2016) and kindergarten math skills revealed predictive value up until high school, even when controlled for other academic skills and family background (Watts et al., 2014). In particular, the growth in math skills between 54 months and first grade appeared to be the strongest predictor of adolescent mathematics. Early numeracy skills (i.e., knowledge about numbers and quantity) have shown to provide the foundation for more complex mathematical abilities and problem solving up to 6 years later (Duncan et al., 2007; Geary et al., 2013). Research into toddlers’ mathematical abilities is emerging though still limited compared to research with preschoolers and beyond. For example, Reikerås and Salomonsen (2017) demonstrated low stability of toddlers’ weak mathematical abilities from age 33 months up to preschool age based on a comprehensive measure (e.g., counting and numbers, space and shape, math language). Only 25% of weak performing children remained weak in math in preschool, whereas the remainder of children was no longer considered weak. Vice versa, it appeared that a new group of weak performing children emerged in preschool, thus showing relatively small persistence of weak math skills in this young age.

During infancy and toddlerhood important developments take place in children’s socio-emotional skills as well, as they learn to gain control over their emotions and regulate their behavior, which foster skills like helping, sharing and cooperating with peers (Bronson, 2000). Socially competent children are more likely to show positive classroom behavior and better peer relations and are less likely to develop psychopathological problems (e.g., internalizing or externalizing problem behavior; Eisenberg, 1991; Raver and Knitzer, 2002; Denham, 2006). Positive social behavior and emotion understanding in preschool have shown to be related to positive social behavior in kindergarten (Nix et al., 2013). Longitudinal studies among 2- and 3-year old children also showed that self-regulation predicted skills at age 60 months (Morgan et al., 2015). Similarly, longitudinal relations were reported for children’s social competence from preschool to kindergarten (Corredor et al., 2017). Altogether, these studies illustrate a potential cascade effect for socio-emotional competence.

In sum, early childhood is a time for major growth in numerous domains, including language, math, and socio-emotional skills. This developmental period can set the stage for subsequent development thus it is important to gain more insight in how these skills develop at an early age and how these skills may potentially be interrelated. For instance, the importance of language skills is not only related to future outcomes in language, but also to the development of skills in other domains (e.g., math or socio-emotional skills), suggesting cross-domain or even reciprocal relations, as will be discussed in the next section.

### Cross-Domain and Reciprocal Relations

A recent paper investigated within- and cross-domain predictors of academic and social trajectories in elementary school highlighting the importance of language skills (Pace et al., 2019). Indeed, language is viewed as an important cornerstone for development in other areas (Salmon et al., 2016) and language forms the foundation for the development of academic skills, including reading and math (e.g., Dickinson et al., 2003; LeFevre et al., 2010; Purpura and Reid, 2016). It is assumed that a larger oral vocabulary reflects a larger lexicon of words and phrases capturing abstract mathematical concepts, which at the same time facilitates the complex understanding of these concepts. Indeed, there is evidence that larger oral vocabulary at age 2 years is predictive of reading and mathematics ability not only at kindergarten entry (Morgan et al., 2015) but also in Grade 6, 10 years after (Bleses et al., 2016). Likewise, preschoolers’ vocabulary was positively associated with their math skills 1 year later (Purpura et al., 2011). LeFevre et al. (2010) illustrated the importance of language skills in kindergarten in predicting math skills 3 years later. Relatedly, language delays have shown to be associated with deficits in math skills among preschoolers and kindergarteners (e.g., Justice et al., 2009; Nelson et al., 2010), although not fully consistent (Aguilar-Mediavilla et al., 2019).

In addition, language supports children to build relationships with significant others, such as peers and teachers, and allows them to express their feelings, desires and wishes and ultimately monitor and regulate their behavior (e.g., Denham et al., 2003; Eisenberg et al., 2005; Fabes et al., 2008). In their review, Salmon et al. (2016) showed the importance of language for socio-emotional development and self-regulation skills. Several studies have illustrated positive relations between children’s language skills and socio-emotional development (e.g., Welsh et al., 2001; Morgan et al., 2015; Vitiello and Williford, 2016). Moreover, several studies have documented comorbidity between toddlers’ language skills and socio-emotional problems (e.g., Carson et al., 1998; Plomin et al., 2002; Horwitz et al., 2003; Morgan et al., 2015), although another study revealed no associations (Rescorla et al., 2007). One study showed that almost half of the 2-year olds with a language delay continued to lag behind 1 year later (Paul et al., 1991). Moreover, these children also lagged behind in their socio-emotional development. Even when they showed language skills in the normal range 1 year later, they continued to show deficits in socio-emotional behavior. Likewise, other studies among toddlers showed that better language receptive and expressive abilities were related to fewer disruptive behaviors (Roberts et al., 2018), and conversely that toddlers lagging behind in expressive vocabulary showed more withdrawn problem behavior compared to typically developing peers (Irwin et al., 2002).

Alternatively, another line of research has investigated the role of socio-emotional skills in other domains of development, such as literacy and math skills. Numerous studies have shown that children’s socio-emotional skills were related to literacy and math skills and academic knowledge (Dobbs et al., 2006; Doctoroff et al., 2006; Bierman et al., 2009; Arnold et al., 2012). In addition, children’s self-regulation skills showed positive relations to math, but not language development (Matthews et al., 2009). Likewise, a few long-term relations were reported between children’s socio-emotional skills at school entry and their reading and math skills across six studies, but the magnitude of these relations was small (Duncan et al., 2007). However, another study reported no impact of kindergarten socio-emotional skills on children’s school achievement related to reading and math up to fifth grade (Claessens et al., 2009).

Also, math skills have shown predictive value in other domains of development. In the aforementioned meta-analysis by Duncan et al. (2007) children’s math skills at school entry were the strongest long-term predictor for reading and math skills. Also, other studies have shown that kindergarten math skills can predict executive function, emergent literacy and reading skills (Claessens et al., 2009; Son et al., 2019). In fact, math skills showed the strongest bidirectional cross-domain associations in kindergarteners’ development with executive function and literacy (Cameron et al., 2019).

In sum, the findings support cross-domain relations across language, math and socio-emotional skills, although the exact nature of these interrelations remains unclear. In order to further disentangle possible genetic and shared environmental factors and investigating causal relations, Trzesniewski et al. (2006) used a longitudinal design with 5-year-old monozygotic and dizygotic twins to study the relation between reading and antisocial behavior. The authors concluded that the majority of the variance occurred through a shared environment and that the associations were bidirectional. The review by Salmon et al. (2016) also mentioned bidirectional relations between language and socio-emotional skills, but foregrounded language as the strongest predictor. Another longitudinal study indeed found the bidirectional relations between vocabulary and socio-emotional skills (Morgan et al., 2015). Children’s socio-emotional skills predicted oral vocabulary at age 24 months, which in turn predicted kindergarten socio-emotional skills. In another study, the cross-lagged relations between children’s language and internalizing and externalizing problem behavior were investigated from age 4.5 to age 7 years (Bornstein et al., 2013). This study only supported relations between language in predicting internalizing behavior, but not the other way around.

To conclude, the current knowledge base has shown cross-domain or even reciprocal relations between language, math and socio-emotional skills, although the majority of studies is limited to only two out of three domains. Additionally, the evidence is not completely consistent. The majority of studies included preschoolers or even older children; thus, less is known about cross-domain associations in toddlerhood.

### Individual Differences and Differential Effects

Research has shown individual differences in children’s language, math and socio-emotional skills, and, relatedly, also in the interrelations between those domains that are associated with child or family background factors. In the current study, we focus on child characteristics. Abundant studies have demonstrated gender differences in children’s language, math and socio-emotional development. Overall, girls appear to outperform boys in language and socio-emotional skills (Eisenberg, 1991; Fenson et al., 1994; Birch and Ladd, 1997; Howes et al., 1998; Bleses et al., 2008; Bierman et al., 2009; Ewing and Taylor, 2009; Matthews et al., 2009; Morgan et al., 2015). However, some studies failed to find gender differences in preschoolers’ vocabulary (Stowe et al., 2000; Bierman et al., 2009), literacy (Doctoroff et al., 2006), academic knowledge (i.e., a combination of literacy and math skills; Bierman et al., 2009) or socio-emotional behavior (Stowe et al., 2000; Doctoroff et al., 2006). Another study focusing on toddlers’ math skills revealed no overall differences between boys and girls but showed that when groups were distributed into quartiles, two-thirds of low-performing children were boys (Salomonsen and Reikerås, 2019).

There is some evidence for differential relations between language, math and socio-emotional development over time between girls and boys. For instance, the relation between pro-social behavior and academic knowledge was stronger for girls than for boys (Bierman et al., 2009). For boys, stronger evidence is available pertaining to socio-emotional problem behavior, such that boys with lower language abilities revealed higher levels of disruptive behavior, but such relation was not found for girls (Stowe et al., 2000). In a similar vein, preschool boys who showed more pro-social behavior scored higher on literacy skills, whereas these relations were not found for girls (Doctoroff et al., 2006). Also, for infants and toddlers a concurrent relation was found between expressive language and social ability, although only for boys (Longobardi et al., 2016). Yet another study involving older children showed that the relation between reading skills and antisocial behavior was stronger for boys (Trzesniewski et al., 2006).

Lastly, differential effects are reported depending on children’s age. For instance, Plomin et al. (2002) showed stronger relations between language delays and behavior problems for older children compared to younger children. One explanation could relate to the fact that language development is not yet stable at a very young age. Literature on so-called “late-talkers” (i.e., the 10% lowest scoring children) shows that they partly catch up (for a review see Rescorla and Lee, 2000; Hawa and Spanoudis, 2014), but there is also evidence of persisting language delays (Paul et al., 1991; Rescorla et al., 2000) or language and reading problems in elementary school (Rescorla, 2002). In another study, looking at language delays at 15, 24, 36, and 54 months, the findings indicated that the percentage of children with delays was the lowest for the 3-year-olds compared to either other group. This lends support to the notion of “late-talkers” and suggests that at least part of these children catch up (Justice et al., 2009). In fact, this study showed that only language delays at age 54 months were predictive of development in other domains, including language, literacy, mathematics, social skills and problem behavior, in kindergarteners, suggesting a timing effect at which stability and predictability of language development is strongest (Justice et al., 2009).

The literature supports individual differences in language, math and socio-emotional skills related to gender and age, although the evidence is not fully consistent. Moreover, there are some indications for differential relations between these set of skills. However, little systematic evidence exists on gender or age differences related to toddlers’ language, math and socio-emotional development or differential associations between these three domains.

### Current Study

The current study aims to add to the existing evidence by investigating language, math language and numeracy skills and socio-emotional skills in young children, as important precursors of school readiness, to enhance our understanding of how these skills are interrelated and identify potential differential associations depending on children’s gender and age. Thus, the research questions are as follows: (i) What are the developmental trajectories of children’s language, math language and numeracy skills and socio-emotional skills? (ii) What are the cross-domain associations between development in children’s language, math language and numeracy skills and socio-emotional skills? (iii) Are these developmental trajectories different depending on children’s age or gender?

## Materials and Methods

### Participants

The current study used data from a randomized controlled trial (RCT) study examining the effectiveness of an intervention focused at enhancing Danish children’s language, math, and socio-emotional skills in toddler classrooms (Bleses et al., 2020). For the current purpose, only data of the control group was used. This concerned a business-as-usual group as compared to an intervention group of teachers that followed professional development and implemented the “Play and Learn” intervention. A total of 44 childcare centers containing 86 classrooms were included, which resulted in a sample of a total of 577 children (49.2% girls) from one-and-a-half until 3 years of age. Children were on average 24.95 months old (*SD* = 4.41, range 18–36 months) and 8.3% of the children were of non-Western background. 26.4% of the children had mothers with a higher education (BA and advanced university education) and 22.9% had fathers with a higher education.

### Procedures

The RCT design was approved by the municipalities. The Danish Data Protection Agency approved the collection and treatment of all data for the project (approval no. j.nr. 2014-54-0822). Data collection took place between September 2015 and June 2016. Teachers completed the assessments on-line.

### Child Measures

Three measures were completed by teachers for each child at two times with approximately 7 months in between. Each of the instruments took on average of about 8–10 min to complete, thus teachers spent about 30 min per child completing the instruments.

#### Language Skills

A teacher-based standardized checklist (CDI-Educator; Bleses et al., 2018b) was developed based on the *MacArthur-Bates Communicative Development Inventories* (Fenson et al., 2007) and a Danish adaptation of a short version of the instrument (Vach et al., 2010) measuring vocabulary and language use. This checklist includes 70 items measuring productive vocabulary divided into nine categories of content (for more information see Bleses et al., 2018b). The Vocabulary summary score was calculated adding up the number of words the child could produce. In addition, there were five questions concerning the child’s use of decontextualized language with respect to objects and actions (e.g., whether the child at any time speaks about earlier episodes and persons, who are not present or about something that will happen in the future). For the Language use summary score, the response categories were converted to points and summed up across the five questions (“not yet” [0], “sometimes” [1] or “often” [2]). Test-retest correlations, with a second measurement made approximately 7 months after the first measurement showed a 0.68 correlation for Vocabulary and 0.54 for Language use. Cronbach’s alpha was high for both scales: 0.98 for vocabulary and 0.88 for Language use. The external concurrent validity was measured for a small subsample of children (*n* = 83–93), indicating moderate correlations between Vocabulary and Language use and Receptive and Expressive One Word Picture Vocabulary test (*r* = between 0.43 and 0.65). For more information on the measurement properties of the instrument is reported in a validation study (Bleses et al., 2018b). Both subscales were used to calculate a mean score for children’s language skills.

#### Math Language and Numeracy Skills

Two aspects of math skills were evaluated with a researcher-developed teacher-based checklist as there was no standardized measure that could be applied to toddlers at that point. The two constructs were primarily based on more general work on children’s math development (Frye et al., 2013) and more specific work on content-specific language that is supportive of math development by Purpura and Reid (2016). The checklist consists of 41 items in total of which 13 items measure children’s early numeracy skills (e.g., saying random numbers, understanding of numbers, using number words) and 28 items evaluate children’s comprehension and use of math language (words for sizes, quantities, shapes, and space; See Supplementary Appendix 1 for the full measure). The response categories were converted to points and summed up across the relevant questions (“not yet” [1], “sometimes” [2], “often” [3] or “always” [4]). Internal consistency indicated excellent reliability for subtest (Cronbach’s alpha of 0.94 and 0.96 for numeracy and math language, respectively). Both subscales were used to calculate a mean score for children’s math skills.

#### Socio-Emotional Skills

A Danish adaptation (Sjoe et al., 2017) of the standardized questionnaire, *Socio-emotional Assessment/Evaluation Measure (SEAM)–Research Edition* (Squires, 2014) was used to assess children’s socio-emotional skills. SEAM includes 10 benchmarks assessing 10 domains (called benchmarks) critical to socio-emotional skills: empathy, healthy interactions, expression of emotions, regulation of socio-emotional responses, cooperation, sharing and engaging, regulation of attention and activity level, independence, self-image, and adaptive skills. SEAM has four response categories and were converted to points: 0 = *not true*, 1 = *rarely true*, 2 = *somewhat true*, and 3 = *very true*. The higher the score, the more positive socio-emotional development. Based on the 10 benchmarks two overall indexes were derived: (1) The Empathy Index measuring the child’s ability to communicate own feelings and to read and understand others’ feelings; and (2) The Self-regulation and Cooperation Index measuring the child’s ability to regulate and cooperate, and the child’s adaptability (Sjoe et al., 2017). The toddler version used in the current study showed good reliability (reliability coefficients ranging from 0.82 to 0.91). Both subscales were used to calculate a mean score for children’s socio-emotional skills.

#### Control Variables

The following control variables are used: gender (1 = boy), age time 1 (in months), time elapsed between time 1 and 2 (in months), ethnicity (1 = non-Western background), maternal and paternal education level as dummy variables (1 = BA degree or higher), and Home Learning Environment (HLE). The HLE is an index based on a total of 11 items (Cronbach’s alpha of 0.74) capturing aspects and activities aimed to promote language and math. It includes among others information on the number of children’s books, the child’s age when first looking at books, parents’ behavior during book reading (pointing and labeling), the content of books (e.g., alphabet, numbers, simple forms) and parents’ behavior when going out and about (pointing to and labeling the environment).

### Analysis Strategy

An autoregressive, cross-lagged path analysis was conducted with mean scores of children’s language, math, and socio-emotional skills while controlling for nesting of children using standard errors adjusted for clustering at the classroom level and the aforementioned control variables using Stata version 15^1^. As our path model only contains observed variables with unidirectional causality (e.g., from language at time 1 to language at time 2), we basically use a set of multiple regressions to estimate the parameters of the model (Kline, 2005). There was some missing data and as recommended, missing data were dealt with by using full information maximum likelihood (FIML) estimation (Enders, 2010), in which the standard errors for the parameter estimates are computed using the complete observed information matrix.

Standardized regression coefficients were used as measures of the effect size with β < 0.10 indicating a small effect, a β of around 0.30 a medium-sized effect and β > 0.50 indicating a large effect (Kline, 2005). In addition, the coefficient of determination (CD) provided by Stata reports the amount of explained variance by the models, thereby indicating how well the model fits the data. A Wald χ^2^ test was used to test whether the full cross-lagged model provided a better fit to the data than the pure autoregressive model. To investigate differences in the associations depending on children’s gender and age, a multi group analysis was conducted by estimating separate models for boys and girls, and for younger and older children. The Likelihood Ratio (LR) χ^2^ test was used to test whether the two cross-lagged structural paths, or the unstandardized regression coefficients, for boys and girls and for younger children (12–24 months) as compared to older children (between 24 and 36 months of age) differed significantly from each other.

## Results

Descriptive statistics and correlations between the individual child measures are provided in Tables 1, 2. The correlations in Table 2 revealed both within- and cross-domain zero-order associations between all child measures across the two time points. There appear moderate to strong concurrent relations between all measures. In addition, children’s non-Western immigrant status was negatively associated with all measures and the quality of the HLE showed small positive correlations.

**TABLE 1:** Descriptive information on children’s language, math, and socio-emotional skills at time 1.

		Mean	SD	Range	*N*
Vocabulary (70 items)	Overall	31.40	20.80	0–70	577
	Boys	28.89	21.33	0–70	293
	Girls	33.98	19.95	0–70	284
Language use (5 items)	Overall	4.47	3.16	0–10	577
	Boys	4.09	3.16	0–10	293
	Girls	4.87	3.10	0–10	284
Math language (28 items)	Overall	43.01	14.58	28–112	574
	Boys	42.26	15.03	28–112	293
	Girls	43.79	14.08	28–112	282
Math (13 items)	Overall	19.00	7.65	13–52	574
	Boys	18.55	7.97	13–52	292
	Girls	19.46	7.30	13–48	282
Empathy (6 domains)	Overall	13.01	4.21	0–18	571
	Boys	12.40	4.19	0–18	289
	Girls	13.63	4.15	0–18	282
Self-regulation/cooperation (4 domains)	Overall	8.54	2.68	0–12	571
	Boys	8.12	2.65	0–12	289
	Girls	8.98	2.68	0–12	282

**TABLE 2:** Correlations between the child measures and background characteristics.

Measure	2	3	4	5	6	7	8	9	10	11	12	13
1. Language 1	0.60***	0.71***	0.60***	0.58***	0.33**	−0.13***	0.59***	–0.03	−0.14***	–0.01	–0.03	0.29***
2. Language 2		0.46***	0.74***	0.49***	0.55***	−0.19***	0.32***	–0.01	−0.16***	–0.03	–0.02	0.22***
3. Math 1			0.55***	0.46***	0.20***	–0.06	0.52***	–0.01	−0.12**	–0.02	–0.02	0.25***
4. Math 2				0.44***	0.45***	−0.11*	0.40***	0.03	−0.08*	–0.02	0.04	0.23***
5. Soc-emo 1					0.55***	−0.17***	0.35***	0.01	−0.11***	–0.02	–0.03	0.13**
6. Soc-emo 2						−0.20***	0.06	0.00	–0.05	–0.01	0.03	0.07
7. Gender							–0.00	0.00	–0.00	–0.08	–0.02	0.00
8. Child age								−0.09*	0.05	–0.08	−0.14**	0.25***
9. Time between waves									0.05	−0.12**	–0.08	0.05
10. Non-Western immigrant										−0.13**	–0.08	–0.07
11. Mat. educ											0.51***	0.05
12. Pat. educ												0.05
13. HLE												

Soc-emo, Socio-emotional; Mat. educ, Maternal education; Pat. educ., Paternal education; HLE, Home Learning Environment. *p < 0.05, **p < 0.01, ***p < 0.001.

Table 3 presents the results of the cross-lagged path analyses. Following the Wald-tests, presented in the table, the cross-lagged models fitted the data better than the autoregressive models. The findings showed moderate to strong stability of children’s language and socio-emotional skills and small stability of children’s math language and numeracy skills, controlled for child and family background variables. Regarding the cross-lagged paths, children’s language skills at time 1 predicted children’s math language and numeracy skills and socio-emotional skills at time 2 both with small effect sizes, but clearly higher for math language and numeracy skills. Likewise, children’s socio-emotional skills appeared predictive of growth in language and math language and numeracy skills at time 2, both with a small effect size. Math skills at time 1 were unrelated to growth in children’s language and socio-emotional skills. For language and math language and numeracy skills, the cross-lagged models explained 41% of the variance, whereas for children’s socio-emotional skills the model explained 32% of the variance.

**TABLE 3:** Standardized regression coefficients of the autoregressive and cross-lagged paths (*N* = 577).

	Language T2	Math T2	Socio-emotional T2
Language time 1 (SE)	0.44*** (0.05)	0.31*** (0.08)	0.16* (0.07)
Math time 1 (SE)	0.06 (0.05)	0.24** (0.07)	−0.04 (0.08)
Socio-emotional time 1 (SE)	0.19*** (0.05)	0.13* (0.06)	0.52*** (0.05)
Gender (1 = boy)	−0.10**	−0.03	−0.11*
Age in months time 1	−0.05	0.06	−0.22**
Time between wave 1 and 2	−0.01	0.05	−0.01
Non-Western immigrant	−0.07	−0.00	0.03
Maternal education (1 = high)	−0.04	−0.04	−0.01
Paternal education (1 = high)	0.01	0.08*	0.00
Home learning environment	0.05	0.04	−0.01
Explained variance (CD)	0.41	0.41	0.32
Wald test χ^2^ (2)	17.77	61.58	7.99
	*p* < 0.001	*p* < 0.001	*p* = 0.02

*p < 0.05, **p < 0.01, ***p < 0.001.

Tables 4, 5 present the findings for boys and girls separately and for younger versus older children. These results revealed some differential relations depending on gender and age. For boys it appeared that initial language skills were predictive of growth in math language and numeracy skills and socio-emotional skills with a medium effect size, whereas for girls these associations were not found. For girls, the results showed that better socio-emotional skills at time 1 were associated with more growth in math language and numeracy skills at time 2 with a small effect. The models for language and math language and numeracy skills explained more variance for boys (48% and 47%, respectively) compared to girls (31% and 38%, respectively). For socio-emotional skills, the explained variance was 34–35% for both boys and girls. The LR test indeed confirmed that the models for language, math language and numeracy skills and socio-emotional differed significantly between boys and girls (see Table 4).

**TABLE 4:** Standardized regression coefficients of the path analyses for boys (*N* = 293) and girls (*N* = 284).

	Language T2		Math T2		Soc-emotional T2	
			
	boy	girl	boy	girl	boy	girl
Language time 1	0.52*** (0.06)	0.34*** (0.09)	0.40*** (0.10)	0.18 (0.10)	0.29** (0.10)	0.07 (0.09)
Math time 1	0.07 (0.05)	0.06 (0.10)	0.18 (0.10)	0.35** (0.11)	0.00 (0.08)	−0.14 (0.10)
Socio-emotional time 1	0.20** (0.08)	0.19** (0.07)	0.13 (0.07)	0.15* (0.07)	0.45*** (0.07)	0.59*** (0.07)
Age in months time 1	−0.09	0.01	0.13*	−0.03	−0.31***	−0.06
Time between wave 1 and 2	−0.04	0.04	0.03	0.08	0.07	−0.10
Non-Western immigrant	−0.04	−0.09	0.04	−0.02	0.13*	−0.07
Maternal education (1 = high)	−0.08	0.00	−0.01	−0.08	−0.02	0.01
Paternal education (1 = high)	0.05	−0.03	0.11	0.06	0.02	−0.01
Home learning environment	0.05	0.04	−0.03	0.08	0.02	−0.07
Explained variance (CD)	0.48	0.31	0.47	0.38	0.35	0.34
LR χ^2^ (64)	124.14 *p* < 0.001		121.59 *p* < 0.0021		141.51 *p* < 0.001	
Model fit (AIC/BIC)	5953.46	5576.83	5983.46	5651.52	5895.20	5421.33
	6188.99	5810.37	6218.99	5885.05	6130.74	5654.86

*p < 0.05, **p < 0.01, ***p < 0.001.

**TABLE 5:** Standardized regression coefficients of the path analyses for younger (*N* = 310) and older (*N* = 267) children.

	Language T2		Math T2		Soc-emotional T2	
			
	young	old	young	old	young	old
Language time 1	0.39*** (0.08)	0.36*** (0.08)	0.34*** (0.09)	0.16 (0.09)	0.15* (0.06)	0.23* (0.11)
Math time 1	0.02 (0.06)	0.12 (0.09)	0.06 (0.07)	0.41*** (0.09)	0.02 (0.06)	−0.28** (0.10)
Socio-emotional time 1	0.20** (0.07)	0.19** (0.07)	0.17* (0.07)	0.11 (0.06)	0.49*** (0.05)	0.53*** (0.09)
Gender	−0.12*	−0.06	−0.08	0.02	−0.09*	−0.14
Age in months time 1	0.07	0.02	0.14*	0.06	−0.14*	−0.15
Time between wave 1 and 2	0.05	−0.06	0.09	0.03	0.02	−0.10
Non-Western immigrant	−0.07	−0.05	0.02	0.02	0.01	0.06
Maternal education (1 = high)	−0.01	−0.06	−0.02	−0.06	0.04	−0.06
Paternal education (1 = high)	−0.03	0.08	0.04	0.14*	−0.09	0.19**
Home learning environment	0.03	0.07	0.01	0.08	−0.04	−0.00
Explained variance (CD)	0.39	0.42	0.35	0.42	0.33	0.43
LR test χ^2^ (81)	147.25 *p* < 0.001		152.53 *p* < 0.001		145.85 *p* < 0.001	
Model fit (AIC/BIC)	6097.10	5444.10	6105.74	5532.30	6164.98	5181.22
	6377.34	5695.20	6385.98	5783.41	6445.22	5432.33

*p < 0.05, **p < 0.01, ***p < 0.001.

There also appeared differential relations depending on children’s age. Language and socio-emotional skills at time 1 was related to younger children’s math language and numeracy skills, whereas these associations were not found for older children. Further, the results revealed that math skills at time 1 were related to less growth in socio-emotional skills for older children, but not for younger children. The explained variance appeared to be slightly higher for older children (between 42 and 43%) compared to younger children (33–39%), especially for children’s socio-emotional skills. The LR tests revealed significant differences between younger (12–24 months) and older (24–36 months) children for all three outcomes (see Table 5).

## Discussion

The current study investigated infants’ and toddlers’ early pre-cursors of school readiness, namely language, math language and numeracy and socio-emotional skills to enhance our understanding of the within- and cross-domain development of these skills and potential differential effects depending on gender and age. It adds to the body of evidence of cross-domain and reciprocal relations in children in preschool and elementary school age and can thus provide additional insights in how these developments take place in infancy and toddlerhood.

The results showed moderate to strong stability of children’s language and socio-emotional development, which is in line with previous research involving toddlers (Lee, 2011; Duff et al., 2015; Morgan et al., 2015; Bornstein et al., 2016) and older children (Whitehurst and Lonigan, 1998; Tomblin et al., 2000; Scarborough, 2001; Welsh et al., 2001; Storch and Whitehurst, 2002; Nix et al., 2013; Bleses et al., 2016; Corredor et al., 2017). However, contrary to research with older children, the current findings revealed lower stability for children’s math skills (Duncan et al., 2007; Clements and Sarama, 2011; Geary et al., 2013; Watts et al., 2014; Nguyen et al., 2016; Purpura and Reid, 2016). One possible explanation could be that these skills are rapidly developing across this age period, but that at a younger age there is larger variability and stability only emerges as children grow older. This is in line with a previous study among toddlers which revealed little stability concerning weak performing children (Reikerås and Salomonsen, 2017). The current multigroup analyses comparing infants and toddlers provides additional support as there were only moderate associations between time 1 and 2 math language and numeracy skills for the youngest children, whereas for older children this association was moderate.

Regarding the cross-domain associations, the current study confirmed previous work as indicated by predictive relations of socio-emotional and language skills for all three domains, with the strongest associations for the latter (Welsh et al., 2001; Dickinson et al., 2003; Dobbs et al., 2006; Doctoroff et al., 2006; Bierman et al., 2009; Matthews et al., 2009; LeFevre et al., 2010; Arnold et al., 2012; Morgan et al., 2015; Purpura and Reid, 2016; Vitiello and Williford, 2016; Pace et al., 2019). Language is viewed as an important gateway for development in other domains (e.g., Salmon et al., 2016). It appeared to be the strongest predictor of children’s math language and numeracy skills at time 2, especially for younger children. This could be explained by the fact that the measure used to assess math skills relied heavily on language competencies (counting, verbalizing words for sizes, quantities, shapes, and space) and suggests that a certain level of general vocabulary skills may be necessary for children to develop more specific math related language. Indeed, for older children math language and numeracy skills were stronger predictors of math language and numeracy development than language skills lending support to this hypothesis. Previous research with preschoolers and kindergarteners highlighted the importance of math-related language over general vocabulary (Purpura and Reid, 2016). There also appeared negative predictions of girls’ math for socio-emotional skill development, whereas the opposite was true for the prediction of math skills. This pattern may suggest that not all skills develop simultaneously and that for girls it seems more beneficial to have socio-emotional skill development precede math skill development. However, more research is warranted to further explore these associations, also in the long run.

Language also appeared to be a stronger predictor for boys’ socio-emotional development, whereas such association was not found for girls. This is in line with other research in which boys’ language abilities have shown to be related to their socio-emotional behavior (Stowe et al., 2000; Longobardi et al., 2016). Some studies have illustrated that boys have poorer socio-emotional skills than girls (Eisenberg, 1991; Howes et al., 1998; Bierman et al., 2009; Ewing and Taylor, 2009; Matthews et al., 2009) and based on the current findings, it may be worthwhile to focus more attention on boys’ language development in addition to socio-emotional competence in order to better support their socio-emotional development.

Contrary to studies with older children, math at time 1 was unrelated to growth in language and socio-emotional development (Duncan et al., 2007; Claessens et al., 2009; Cameron et al., 2019; Son et al., 2019), which could possibly be explained by the relatively low levels of math language and numeracy skills at time 1. For 2- and 3- years old children it even appeared that higher math skills at time 1 was associated with less growth in their socio-emotional skills, which was not the case for younger children. This may indicate that for 2- and 3-year olds, math skills do not develop in conjunction with socio-emotional skills but rather constitute a separated line of development. The research evidence on cross-domain development in toddlers is only emerging, especially regarding the role of math, therefore the current study only allows for drawing tentative conclusions. Further research is warranted to substantiate more solid conclusions.

In line with previous research there appeared to be bidirectional relations between children’s socio-emotional and language skills (Welsh et al., 2001; Morgan et al., 2015; Salmon et al., 2016; Vitiello and Williford, 2016). Also, socio-emotional skills appeared to be associated with growth in math skills, in line with previous research in older children (Duncan et al., 2007; Matthews et al., 2009). In the current study, the concept of socio-emotional development was a rather broad construct, including aspects of self-regulation and the regulation of attention and activity level, which could explain these associations as previous research particularly highlighted the role of self-regulation and executive function in predicting language and math development in older children (e.g., Blair and Razza, 2007; Matthews et al., 2009).

The current findings could have some implications for practice, especially in Early Childhood Education (ECE) provisions. Given that children’s language and socio-emotional skills were the strongest predictors across domains, this points to the need to address these foundational skills in curricula and daily play and activities for young children. This would require a concerted effort to include both a focus on providing a rich language environment while at the same time fostering children’s opportunities to develop socio-emotional skills in interactions with peers. Recent evidence on skill-based curricula for toddlers indeed revealed substantial effects on language, math and socio-emotional skills development (Landry et al., 2014; Bleses et al., 2020) supporting the need to use explicit and sequenced provision of play and activities to boost children’s school readiness.

The current study suffers from a number of limitations. Firstly, all child measures relied on teacher reports. Although concerns have been expressed about the validity of providing estimates of children’s development, it is a common methodology in large-scale research involving the assessment of children’s development (e.g., the National Center for Early Development and Learning’s Multi-State Study of Pre-Kindergarten, the joint National Center for Early Development and Learning–National Institute for Early Education Research State-Wide Early Education Programs Study, and the Twins Early Development Study) and a number of studies have shown that teacher reports were valid and reliable measures of children’s behavioral (Bishop et al., 2003) and academic skills (National Center for Education Statistics [NCES], 2002; Justice et al., 2009). Especially concerning children’s math skills, it may be that teacher ratings underestimated children’s skills as they may not have been sufficiently aware of their skill level. Nonetheless, future research is needed to investigate whether the findings can be replicated when using test-based measures. Second, the current study is based on two measurement points within a time period of 1 year. Longitudinal research from an early age is warranted to corroborate the current findings. Note, however, that the current study included children from 18 to 36 months of age, allowing for age group comparisons that indeed revealed different within- and cross-domain associations for infants and toddlers.

In conclusion, the current study investigated the development of important pre-cursors of children’s school readiness defined as a broad construct encompassing socio-emotional, language and math skills, which are known to be important for success in preschool, kindergarten and beyond. The period between 18 and 36 months of age showed to be an important age period for the development of these skills and showed some different patterns of development as compared to the preschool period. Children’s language and socio-emotional skills appeared to be the most important skills in predicting other aspects of school readiness, whereas the results for math skills at this young age appear less consistent. This has important implications for ECE practice aimed at fostering broad development in young children to provide them with the best start in life.

## Data Availability Statement

Because the data include confidential information on individual citizens, they are not publicly available but placed on a secured server hosted by Statistics Denmark. Independent researchers can apply to Statistics Denmark for access.

## Ethics Statement

The RCT design was approved by the five municipalities. The Danish Data Protection Agency approved the collection and treatment of all data for the project (approval no. j.nr. 2014-54-0822). All teachers and parents were informed about the RCT and data collection activities, with materials provided in five different languages, and were assured that all data would be treated anonymously and confidentially.

## Author Contributions

PS wrote the manuscript, analyzed the data, and interpreted the results. DB was principal investigator, designed the study, and revised the manuscript. PJ analyzed the data and revised the manuscript. All authors contributed to the article and approved the submitted version.

## Conflict of Interest

The authors declare that the research was conducted in the absence of any commercial or financial relationships that could be construed as a potential conflict of interest.

## Footnotes

1 Analyses were also conducted using the numeracy skills measure only to evaluate the relations without the math language component. The results of these analyses, which confirm the overall pattern of results including both math measures, are presented in Supplementary Appendix 2.
