# Supplementary material for: Infants’ and Toddlers’ Language, Math and Socio-Emotional Development: Evidence for Reciprocal Relations and Differential Gender and Age Effects
Source: Front Psychol. 2020 Nov 23;11:580297. doi: 10.3389/fpsyg.2020.580297 (PMC7732521; doi:10.3389/fpsyg.2020.580297)
Supplement: Supplementary file 1 [file Table_1.docx]

**Appendix 1: Overview of Math Items**

| **Subscale** | **Item** |
| --- | --- |
| Math | Can the child say any random numbers regardless of whether they are used to count (e.g., I have a thousand pictures)? |
| Math | Does the child perceive the number of objects up to three without counting (e.g., three cars in a picture)? |
| Math | Does the child show understanding of numbers by using his/her fingers when you ask, for example: How old are you? (it does not matter if it is the correct number the child shows with his fingers)? |
| Math | Does the child sometimes say a series of numbers such as 3, 4, 5, for example, when the child places plates on the table? (it does not matter what numbers it is)? |
| Math | Does the child hand you the right number of objects (between 1 and 3) when you say, for example: Hand me two bricks? |
| Math | Does the child use some number words correctly, i.e., say two when there are two objects? |
| Math | Can the child connect the correct number with a quantity (up to 3) e.g., in puzzles or in books where 2 cats and the 2 number belong together? |
| Math | Does the child use number words from 1 to 5 when describing one or more objects, e.g. 2 spoons? (it does not matter if it is the correct number) |
| Math | Does the child ask questions about numbers and quantities? (e.g., does the child ask how old someone is or how many teddy bears there are?) |
| Math | Does the child answer correctly: What comes after 1? |
| Math | Does the child count up to five objects correctly by pointing to each object and at the same time saying the correct thing |
| Math | Does the child answer correctly: What comes after 2? |
| Math | Does the child answer correctly when you ask:? Which is bigger: 3 or 4? |
| Math language | Can the child designate a square when the child is presented with different shapes (e.g., circles, triangles and squares)? |
| Math language | Can the child designate a circle or "the round one", when the child is presented with different shapes (e.g., circles, triangles and squares)? |
| Math language | Can the child designate a triangle when the child is presented with different shapes (e.g., circles, triangles and squares)? |
| Math language | Can the child name a square when you point to a square and ask the child what it is called? |
| Math language | Can the child name a circle / "a round one", when you point to a circle and ask the child what it is called? |
| Math language | Can the child name a triangle when you point to a triangle and ask the child what it is called? |
| Math language | Does the child understand words that describe "quantity"? (e.g., many or few)? |
| Math language | Has the child itself started using words that describe "quantity" (e.g., many or few)? |
| Math language | Does the child understand words that describe size as big and small (e.g., I want the little teddy bear)? |
| Math language | Has the child itself started using words that describe size as big and small (eg I want the little teddy bear)? |
| Math language | Does the child understand words that describe temperature as hot and cold (e.g., the ice is cold)? |
| Math language | Has the child itself started using words that describe temperature as hot and cold (e.g., the ice is cold)? |
| Math language | Does the child understand words as more or less when comparing two groups of objects? |
| Math language | Has the child itself begun to use words as more or less when comparing two groups of objects? |
| Math language | Does the child understand words that describe weight as heavy and light (e.g., my bucket is heavy)? |
| Math language | Has the child itself started using words that describe weight as heavy and light (e.g., my bucket is heavy)? |
| Math language | Does the child understand words that describe speed as fast and slow (e.g., my bike is fast)? |
| Math language | Has the child itself started using words that describe speed as fast and slow (e.g., my bike is fast)? |
| Math language | Does the child understand words that describe length as short and long (e.g., I want the long stick)? |
| Math language | Has the child itself started using words that describe length as short and long (e.g., I want the long stick)? |
| Math language | Does the child understand words that describe time like yesterday and tomorrow (e.g., tomorrow I have to play with Sigrid)? |
| Math language | Has the child itself started to use words that describe time like yesterday and tomorrow (e.g., tomorrow I have to play with Sigrid)? |
| Math language | Does the child understand words like bigger, biggest, smallest that compare the sizes of objects (e.g., I want a bigger ice cream)? |
| Math language | Has the child itself started using words bigger, biggest, smallest that compare the sizes of objects (e.g., I want a bigger ice cream)? |
| Math language | Can the child follow directions such as crawling under the table or jumping over the bench? |
| Math language | Can the child follow directions such as standing next to the table or hiding behind the tree? |
| Math language | Does the child use words, that describe location in space (e.g., above or below)? |
| Math language | Does the child use words, that describe location in space (e.g., next to or behind)? |

**Appendix 2 Results of the Main and Subgroup Analyses with only the Math Numbers Subscale**

*Standardized Regression Coefficients of the Autoregressive and Cross-Lagged Paths (N=577)*

|  | Language T2 | Math T2 | Socio-emotional T2 |
| --- | --- | --- | --- |
| Language time 1 (SE) | .45***  (.05) | .33*** (.08) | .18**  (.07) |
| Math time 1 (SE) | .04  (.05) | .16**  (.06) | -.08  (.08) |
| Socio-emotional time 1 (SE) | .20*** (.05) | .13* (.06) | .52***  (.05) |
| Gender (1=boy) | -.10** | -.03 | -.11* |
| Age in months time 1 | -.05 | .09* | -.22** |
| Time between wave 1 and 2 | -.01 | .05 | -.01 |
| Non-Western immigrant | -.07 | .00 | .04 |
| Maternal education (1=high) | -.04 | -.02 | -.01 |
| Paternal education (1=high) | .01 | .08 | .00 |
| Home learning environment | .06 | .03 | -.01 |
|  |  |  |  |
| Explained variance (CD) | .40 | .37 | .32 |
| Wald test χ^2^ (2) | 17.08 *p* < .001 | 56.00 *p* < .001 | 9.39 *p* = .02 |

* *p* < .05, ** *p* < .01, *** *p* < .001

*Standardized Regression Coefficients of the Path Analyses for Boys (n=293) and Girls (n=284)*

|  | Language T2 | | Math T2 | | Soc-emotional T2 | |
| --- | --- | --- | --- | --- | --- | --- |
|  | boy | girl | boy | girl | boy | girl |
| Language time 1 | .53*** (.06) | .37***  (.08) | .39*** (.10) | .26*** (.10) | .231* (.10) | .07  (.08) |
| Math time 1 | .06 (.04) | .00 (.08) | .14 (.09) | .21* (.10) | -.04 (.10) | -.15 (.08) |
| Socio-emotional time 1 | .21** (.08) | .19** (.07) | .12 (.07) | .15 (.08) | .45*** (.07) | .58*** (.07) |
| Age in months time 1 | -.08 | .02 | .16* | .01 | -.31*** | -.06 |
| Time between wave 1 and 2 | -.04 | .04 | .01 | .10 | .07 | -.09 |
| Non-Western immigrant | -.04 | -.09 | .05 | -.03 | .13* | -.07 |
| Maternal education (1=high) | -.08 | .00 | -.00 | -.04 | -.03 | .01 |
| Paternal education (1=high) | .05 | -.03 | .09 | .06 | .02 | -.01 |
| Home learning environment | .05 | .04 | -.04 | .08 | .02 | -.08 |
|  |  |  |  |  |  |  |
| Explained variance (CD) | .48 | .31 | .42 | .34 | .35 | .34 |
| LR χ^2^ (64) | 114.93  *p* < .001 | | 106.83 *p* < .0021 | | 132.06 *p* < .001 | |

* *p* < .05, ** *p* < .01, *** *p* < .001

*Standardized Regression Coefficients of the Path Analyses for Younger (n = 310) and Older (n = 267) Children*

|  | Language T2 | | Math T2 | | Soc-emotional T2 | |
| --- | --- | --- | --- | --- | --- | --- |
|  | young | old | young | old | young | old |
| Language time 1 | .39*** (.08) | .39*** (.08) | .33*** (.10) | .21* (.09) | .15* (.06) | .29* (.09) |
| Math time 1 | .02 (.05) | .08 (.07) | .01 (.06) | .32*** (.09) | .01 (.05) | -.39** (.09) |
| Socio-emotional time 1 | .20** (.07) | .19** (.07) | .16* (.07) | .11 (.06) | .49*** (.05) | .53*** (.08) |
| Gender | -.12* | -.06 | -.07 | .03 | -.09* | -.15 |
| Age in months time 1 | .07 | .02 | .16* | .09 | -.14* | -.18 |
| Time between wave 1 and 2 | .05 | -.06 | .07 | .05 | .02 | -.11 |
| Non-Western immigrant | -.07 | -.05 | .05 | -.00 | .01 | .07 |
| Maternal education (1=high) | -.01 | -.06 | .01 | -.05 | .04 | -.08 |
| Paternal education (1=high) | -.03 | .08 | .06 | .11 | -.09 | .18** |
| Home learning environment | .03 | .08 | .03 | .05 | -.03 | -.02 |
|  |  |  |  |  |  |  |
| Explained variance (CD) | .39 | .42 | .31 | .37 | .33 | .47 |
| LR test χ^2^ (81) | 146.15 *p* < .001 | | 146.23 *p* < .001 | | 150.39 *p* < .001 | |

* *p* < .05, ** *p* < .01, *** *p* < .001
